# Effectiveness of Chinese patent medicine in the treatment of influenza: A protocol for systematic review and meta-analysis

**DOI:** 10.1097/MD.0000000000027766

**Published:** 2021-11-19

**Authors:** Kang Liu, Yongkang Zhang, Xin Song

**Affiliations:** a School of Traditional Chinese Medicine, Jiangxi University of Traditional Chinese Medicine, China; b School of Traditional Chinese Medicine, Shandong University of Traditional Chinese Medicine, China.

**Keywords:** Chinese patent medicines, influenza, meta-analysis, systematic review

## Abstract

**Background:**

Western medicine mainly uses antiviral drugs to treat influenza. However, anti-influenza virus drugs have been reported to have high levels of drug resistance and varying degrees of adverse reactions. In addition, Western medicine uses vaccination to prevent influenza, but vaccination cannot ensure complete prevention. Vaccines have a certain lag and cannot prevent the constantly mutating influenza virus. At present, there are still certain limitations in the prevention of influenza. In recent years, traditional Chinese medicine has been used more and more widely in the prevention and treatment of influenza and improvement of influenza-like symptoms, and related clinical efficacy control studies have reached a certain number. Therefore, the purpose of this study is to systematically evaluate the effectiveness of Chinese patent medicine in the treatment of influenza.

**Methods:**

Computer search of PubMed, Cochrane Library, Embase, CNKI, Wangfang and VIP database, search for randomized controlled trials of Chinese patent medicines therapy on influenza, the search time limit is to build the database until October 2021. Two researchers screened the retrieved literature and collected relevant patient information and data. The final included literature was meta analyzed by Rev man5.4 software.

**Results:**

The effectiveness and safety of Chinese patent medicines in the treatment of patients with influenza will be systematically evaluated.

**Conclusion:**

Systematic collection and analysis of clinical randomized controlled trials of Chinese patent medicines for the treatment of influenza, with a view to providing basic information for clinical decision-making and related research.

**Registration number:**

INPLASY2021100064 (https://inplasy.com/inplasy-2021- 10-0064/).

## 1 Introduction

Influenza is an acute respiratory infection caused by RNA virus infection of the Myxoviridae family. It is mainly transmitted through droplets and contact and the clinical manifestations are sudden high fever, muscle aches, fatigue, and mild respiratory symptoms.^[1]^ Influenza viruses are significant human respiratory pathogens that cause both seasonal, endemic infections and periodic, unpredictable pandemics.^[2]^ As influenza virus is extremely contagious and humans are generally susceptible to it, it is highly prone to pandemics and is one of the important diseases for prevention and control of various countries. According to a study by the World Health Organization, the annual seasonal epidemic of influenza will cause 5% to 10% of the global population to be infected, resulting in 3 million to 5 million hospitalized cases and 290,000 to 650,000 deaths.^[3]^ Influenza virus antigens are changeable and seasonally prevalent, and are generally susceptible to all age groups. Among them, it is most common in patients with chronic underlying diseases and the elderly group, and can lead to a higher mortality rate, which has a huge impact on society and the population.

In addition, complications of influenza are more common, especially in the elderly, children, pregnant women, postpartum women, and people with underlying diseases,^[4]^ among which other respiratory diseases are more common, such as bronchitis and pneumonia, Otitis media, sinusitis, etc, while extrapulmonary complications are relatively rare, such as present encephalitis, myelitis, meningitis, seizures, toxic shock syndrome, etc, neurological complications are more common in children.^[5]^ Pre-existing chronic diseases can also appear or worsen, such as asthma, chronic obstructive pulmonary disease, and congestive heart failure.

In recent years, various subtypes of influenza have occurred frequently, Western medicine mainly adopts symptomatic treatments such as antipyretic and analgesic treatment. However, drugs often cause bone marrow suppression, damage liver and kidney function, and can also cause gastric mucosal damage. In addition, coupled with the lag in understanding the cause of the disease in modern Western medicine, it is difficult to meet clinical needs. The basic idea of “differentiation and treatment” of traditional Chinese medicine (TCM) can provide symptomatic treatment before clarifying the causative factors, solve the shortcomings of Western medicine treatment, and achieve better results.

For the treatment of influenza, the antiviral drugs recommended by the Health Commission's 2018 guidelines are oseltamivir, zanamivir, and peramivir. However, based on the results of meta-analysis, the role of oseltamivir in preventing and treating influenza is controversial,^[6]^ and the resistance rate of oseltamivir to influenza virus is also increasing. TCM is effective in the treatment of influenza, it can significantly alleviate the clinical symptoms of influenza patients, and promote the recovery of the disease, thereby shortening the course of disease. From SARSA, the outbreak of influenza in 2009 to the epidemic fight of COVID-19, Chinese medicine treatment has been practically verified. TCM has unique advantages in the prevention and treatment of influenza. While anti-virus, Chinese medicine can reduce fever, reduce inflammation, and regulate the body's immunity, thereby controlling the spread of inflammation and quickly alleviating clinical symptoms. At present, there have been a large number of studies on the mechanism of Chinese medicine against influenza virus, which are mainly reflected in regulating the immune function of the body, regulating the apoptosis and autophagy induced by influenza virus, regulating the oxidative stress response caused by influenza virus, and inhibiting the invasion and release of influenza virus.^[7–9]^ At the same time, it can interfere with influenza virus RNA polymerase activity and regulate cell pathways. In addition, due to the compatibility of traditional Chinese medicines and the diverse activities of traditional Chinese medicines, viruses rarely develop resistance to traditional Chinese medicines.

In recent years, many effective Chinese medicines and compound prescriptions have been made into Chinese patent medicines, which are widely used in clinical practice and have significant effects. This study conducted a comprehensive summary and analysis of clinical studies on the treatment of influenza with Chinese patent medicines, with a view to providing references for clinical diagnosis and treatment practices and related research.

## 2 Methods

The protocol will be reported strictly according to the Preferred Reporting Items for Systematic Reviews and Meta-Analyses Protocols (PRISMA-P) statement. This study has been registered in the INPLASY website (registration number is INPLASY2021100064).

### 2.1 Eligibility criteria of inclusion of studies

#### 2.1.1 Types of studies

We will include all randomized controlled trials that study the treatment of influenza in the Chinese patent medicines. If the following conditions are included, they will be excluded.

1. Intervention measures are non-oral proprietary Chinese medicines, such as the use of Chinese herbal decoctions or external use of Chinese medicines.
2. Research where the data are abnormal or cannot be extracted.
3. For documents published by the same author or team but with the same research results, select one of them for inclusion.
4. The usage and dosage of the drug do not conform to the drug instructions.
5. Non-randomized controlled trials (RCT) research: such as experts and professors’ personal experience summaries, reviews, in vitro experiments, animal experiments, pharmacological experiments, case reports, conference papers, etc.

#### 2.1.2 Types of participants

Western medicine diagnostic standards refer to the “Influenza Diagnosis and Treatment Plan (2019 Edition)” issued by the National Health Commission.

The clinically diagnosed case has an epidemiological history and other diseases that cause influenza-like symptoms are excluded.

Confirm that the diagnosed case has clinical manifestations of influenza and meets one of the following conditions: influenza virus nucleic acid test is positive, or influenza virus antigen test is positive. Influenza-like cases refer to acute onset (within 2 days), fever (body temperature ≥38 °C), accompanied by cough or sore throat, lack of other laboratory judgments. During the influenza season, the accuracy of using influenza-like cases as clinical diagnosis can reach 70% to 80%.

#### 2.1.3 Types of interventions

The test group is oseltamivir combined with a Chinese patent medicine (such as Banlangen granules, Kanggan granules, Lianhua Qingwen granules), the control group is oseltamivir monotherapy. Other interventions (cough medicine or antipyretics are allowed, etc) consistent between the 2 groups.

#### 2.1.4 Types of outcome measures

Including one of the following: antipyretic relief time (recording the patient's body temperature during the test until the temperature is normal or falling below 37.2 °C, and no longer rebounds). Cough relief time (after the patient takes the medicine until the cough symptoms are relieved time). C-reaction protein (C-reaction protein, CRP) decline. Effective rate (healed: symptoms disappear after treatment and body temperature returns to normal. Markedly effective: most symptoms disappear after treatment and body temperature returns to normal. Effective: symptoms after treatment part of it disappeared, and the body temperature was lower than before. Ineffective: the symptoms almost did not disappear after treatment, and the body temperature did not drop or rise). Effective rate = (healed + markedly effective + effective)/total number of cases. Adverse reactions.

### 2.2 Electronic searches

Computer search of Chinese databases: CNKI, Wanfang, VIP and SinoMed, English databases: PubMed, EMbase, and Cochrane Library. Select the advanced search to find the literature on randomized controlled trials of Chinese patent medicines in the treatment of influenza with the period from the establishment of the database to October 2021. In addition, reference documents are traced to supplement relevant documents to minimize omissions. The search terms: “influenza” OR “Chinese patent medicines” OR “traditional Chinese medicine” OR “oseltamivi” AND “randomized controlled trial” OR “RCT.”

### 2.3 Data collection and analysis

#### 2.3.1 Literature screening

Use Note Express3.2.0 software to import documents in batches. First read the titles and abstracts of the articles, eliminate documents that obviously do not meet the inclusion criteria, and then read the full text, eliminate duplicate documents, and finally include relevant documents. Two researchers independently selected and checked documents that met the requirements based on the above inclusion criteria. If there are disputes in the selection of documents, a third party will independently judge the documents that have disagreements, and then negotiate and form a unified opinion.

#### 2.3.2 Data extraction

**Figure 1:**
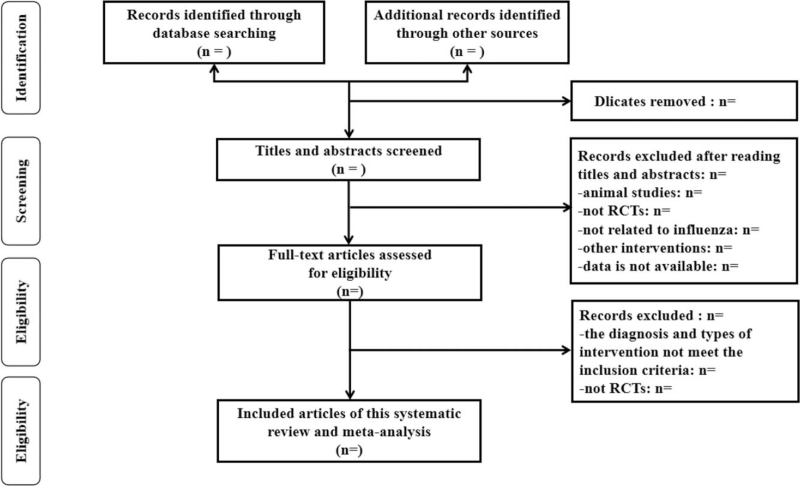
The research flowchart.

Record the relevant research methods in the literature in detail, including whether randomized controlled studies and blinding methods are used, the follow-up status of the research subjects during the research process, whether they are lost to follow-up and the reasons for the loss, record the name of the author, the specific publication year of the article, and the subjects sex, course of treatment and intervention measures taken, etc. Record the outcome and measure related indicators, including fever reduction rate, fever reduction time, cough relief time, headache relief time, muscle sore relief time, nucleic acid conversion rate, and adverse reaction rate. The flowchart will be demonstrated in Fig. 1.

#### 2.3.3 Assessment of risk bias

The RCTs of the included studies were evaluated according to the risk of bias assessment tool in the Cochrane Handbook. Including the following 7 aspects: random method, random allocation plan concealment; selective reporting, whether to blind the research subjects and researchers, whether the data is complete, blind evaluation of the research outcome, other sources that affect the authenticity. According to the content of the included literature, the risk of bias is divided into 3 levels: low-risk, uncertain, and high-risk.

#### 2.3.4 Statistical methods

Meta-analysis uses Revman 5.4 software (Cochrane Collaboration) to analyze data. Binary variables use odds ratio (OR) as the effect size, and 95% confidence interval (CI) is used to express. Continuous variables use mean difference (MD) is used as the effect size and expressed with 95% CI. *P* < .05 considered the difference to be statistically significant, *P* > .05 considered the difference not to be statistically significant. *I*^2^ is used to assess the heterogeneity of the included literature. If the results suggest that *I*^2^ ≤ 50%, indicating that the heterogeneity between the studies is small, the meta-analysis uses a fixed-effects model to conduct a comprehensive analysis of the data. If *I*^*2*^ > 50%, it indicates that the heterogeneity between the studies is large. At this time, it is necessary to eliminate the included studies one by one for sensitivity analysis or perform subgroup analysis according to different methods to eliminate the influence of heterogeneity between the literature. At this time, the meta-analysis adopts the random effects model for data analysis, and finally observe whether the results of the meta-analysis are stable.

#### 2.3.5 Assessment of reporting biases

Draw a correction-comparison funnel chart for the outcome indicators of the included studies >10, observe the symmetrical distribution of the funnel chart scatter points and whether they fall out, to judge whether there is a publication bias or a small sample effect in this study.^[10]^

#### 2.3.6 Assessment of heterogeneity and sensitivity analysis

If the heterogeneity is large, it is necessary to further specifically explore the source of the heterogeneity from the clinical heterogeneity and methodological heterogeneity of the literature, and use sensitivity analysis or subgroup analysis to find the factors that affect the heterogeneity. The source of heterogeneity was discussed through subgroup analysis and sensitivity analysis. Subgroup analysis based on grouping included treatment process, control group intervention measures, patient grouping plan, and so on. If the heterogeneity or the source of heterogeneity cannot be determined when the heterogeneity is not too large, only descriptive analysis will be carried out.

### 2.4 Ethics and dissemination

Due to the agreement of the systematic review and meta-analysis in this article, all data in this study are from published studies and do not involve patient personal information, so ethics committee approval is not required. The results of this research will be distributed to peer-reviewed journals and published in relevant conferences.

## 3 Discussion

Influenza is a contagious acute respiratory disease caused by influenza virus. Influenza has a rapid onset and can cause pandemics in various degrees around the world. It poses a serious threat to human safety, and its economic losses are among the most relevant infectious diseases,^[11]^ and it is also an infectious disease subject to global surveillance.^[12]^ Therefore, it is very effective to explore a safe and effective way to treat influenza. In recent years, a variety of Chinese patent medicines have been developed for the treatment of influenza. This study systematically evaluated the treatment of influenza by Chinese patent medicine, aiming to compare the efficacy and safety of proprietary Chinese medicines and antiviral drugs alone, so as to provide reference for clinical medication selection.

At present, there is a lack of high-quality, multicenter, large-sample randomized controlled trials of Chinese patent medicines for the treatment of influenza as reliable evidence-based evidence. It is hoped that relevant measures will be taken to improve the quality of design, implementation, and reporting of clinical trials of Chinese medicine. In response to the above problems, the following suggestions are made: before the trial is launched, the trial design and implementation personnel should strengthen the study of evidence-based methodology, and strengthen training and dissemination. When the trial design, strictly follow the clinical trial report plan SPIRIT statement^[13]^ to formulate the trial plan, and pay attention to registration, approval, informed consent, sample size estimation, outcome indicators and other key links, standardize the design of clinical research plans. During the implementation of the trial, strictly control the implementation process of the trial, and regularly conduct quality control. After the completion of the trial, the clinical research should strictly follow randomized controlled trial report specification CONSORT statement and STRICTA list statement are reported to standardize the report.

## Author contributions

**Conceptualization:** Kang Liu, Yongkang Zhang, Xin Song.

**Data curation:** Yongkang Zhang, Xin Song.

**Formal analysis:** Kang Liu, Kang Liu, Xin Song.

**Project administration:** Yongkang Zhang, Xin Song.

**Supervision:** Kang Liu.

**Writing – review & editing:** Kang Liu, Yongkang Zhang.
